# Boosting brain connectome classification accuracy in Alzheimer's disease using higher-order singular value decomposition

**DOI:** 10.3389/fnins.2015.00257

**Published:** 2015-07-24

**Authors:** Liang Zhan, Yashu Liu, Yalin Wang, Jiayu Zhou, Neda Jahanshad, Jieping Ye, Paul M. Thompson, Alzheimer's Disease Neuroimaging Initiative (ADNI)

**Affiliations:** ^1^Imaging Genetics Center, Keck School of Medicine, University of Southern CaliforniaMarina del Rey, CA, USA; ^2^School of Computing, Informatics, and Decision Systems Engineering, Arizona State UniversityTempe, AZ, USA; ^3^Department of Computational Medicine and Bioinformatics, University of MichiganAnn Arbor, MI, USA; ^4^Department of Electrical Engineering and Computer Science, University of MichiganAnn Arbor, MI, USA

**Keywords:** Alzheimer's disease, mild cognitive impairment, diffusion MRI, connectome, high-order SVD, classification

## Abstract

Alzheimer's disease (AD) is a progressive brain disease. Accurate detection of AD and its prodromal stage, mild cognitive impairment (MCI), are crucial. There is also a growing interest in identifying brain imaging biomarkers that help to automatically differentiate stages of Alzheimer's disease. Here, we focused on brain structural networks computed from diffusion MRI and proposed a new feature extraction and classification framework based on higher order singular value decomposition and sparse logistic regression. In tests on publicly available data from the Alzheimer's Disease Neuroimaging Initiative, our proposed framework showed promise in detecting brain network differences that help in classifying different stages of Alzheimer's disease.

## Introduction

Alzheimer's disease (AD) is a chronic neurodegenerative disease that involves the accumulation of amyloid plaques and neurofibrillary tangles in the brain. The most common early symptom is difficulty remembering recent events (short-term memory loss). As the disease advances, symptoms often include problems with language, altered affect, disorientation, lack of motivation, problems with self-care, and behavioral abnormalities (Burns, 2009; Burns and Iliffe, 2009). As a patient's condition declines, they may withdraw from family and society. Gradually, more and more bodily functions are lost, ultimately leading to death. Although the speed of progression varies, the average life expectancy following diagnosis is 3–9 years (Querfurth and LaFerla, 2010; Todd et al., 2013). AD has a typical pattern of progression, with anatomical changes that correspond to the types and severity of symptoms. The symptoms, the order in which they appear, and the duration of each clinical stage vary from person to person. Disease progression can be divided into three main stages: normal controls (NC), mild cognitive impairment (MCI) and AD. All of these classifications are defined clinically based on behavioral and cognitive assessments, and although a person with MCI has elevated risk of developing AD, many people with MCI remain stable for some time or may develop other degenerative conditions pathologically distinct from AD, such vascular dementia or fronto-temporal dementia.

NC represents the subset of the population who are aging normally, and do not have sufficiently severe symptoms to be considered cognitively impaired. MCI involves cognitive impairments, but at a level that is not significant enough to interfere with a person's daily activities (Petersen et al., 1999). MCI is often a transitional stage between normal aging and dementia: every year, around 10–15% of people with MCI progress to probable AD (Grundman et al., 2004). However, not all people with MCI deteriorate cognitively and some even improve. Effective and accurate diagnosis of Alzheimer's disease and its prodromal stage, MCI, are crucial for drug trials, given the urgent need for treatments to resist or slow disease progression.

Many neuroimaging studies have used anatomical measures derived from T1-weighted brain MRI, such as cortical thickness, and volumetric or shape measures of subregions of the brain, to differentiate AD or MCI from NC (Fan et al., 2008; Hua et al., 2008a,b; Gerardin et al., 2009; Magnin et al., 2009; Hua et al., 2010; Cuingnet et al., 2011; Westman et al., 2011; Hua et al., 2013; Gutman et al., 2015).

Moreover, measures derived from functional imaging or cerebrospinal fluid (CSF) assays have also been used to help classify individuals with cognitive impairment vs. healthy controls (De Santi et al., 2001; Morris et al., 2001; Bouwman et al., 2007; Mattsson et al., 2009; Shaw et al., 2009; Fjell et al., 2010). Diffusion weighted MRI is a non-invasive imaging technique that can provide clinical information on white matter integrity in a variety of diseases, such as schizophrenia (Zalesky et al., 2011), autism (Lewis et al., 2014), traumatic brain injury (Dennis et al., 2015b), and even in genetics (Jin et al., 2011, 2013) and sex difference (Jahanshad et al., 2011). The white matter integrity can be analyzed with both the tract-based analysis such as tract-based spatial statistics (Smith et al., 2006), fiber clustering (Jin et al., 2012, 2014), and the parcellation-based connectome analysis (Toga et al., 2012).

In particular, many studies have used diffusion-weighted MRI (DWI) to study AD and MCI. Demirhan et al. (2015) studied the added value of diffusion tensor derived measures, over and above structural MRI, and showed they provided added diagnostic accuracy for classification of disease stages (Demirhan et al., 2015). Nir et al. (2013) found that standard diffusion tensor derived measures were strongly correlated with several clinical ratings that are widely-used in AD research (MMSE, CDR-sob, and ADAS-cog) (Nir et al., 2013). When effect sizes were ranked, *mean diffusivity* (MD) measures tended to outperform fractional anisotropy (FA) measures for detecting group differences in tracts that pass through the temporal lobes and the left hippocampal component of the Cingulum. Diffusivity measures tended to detect the more subtle differences in MCI, even when comparisons of FA measures did not. Jin et al. (2015) also used various diffusion-derived measures to relate fornix degeneration with cognitive decline. MD was also shown to be more sensitive to group differences among AD, MCI, and normal controls than FA (Jin et al., 2015).

Several studies used the ADNI DWI scans to compute structural connectivity measures, including measures of the brain's network properties. Li et al. (2013) proposed a spectral diffusional connectivity framework to explore the connectivity deficit in AD. Li et al. (2013) The framework was based on studying the eigenvalues of the Laplacian matrix of the diffusion tensor field at the voxel level. The peaks of the diffusional connectivity spectra were shifted in the AD group versus the normal controls. Prasad et al. (2015) ranked several connectivity measures, to see which ones best distinguished AD from normal aging (Prasad et al., 2015). Graph-based network measures—such as small-world properties, clustering, and modularity—helped in differentiating diagnostic subgroups relative to just using the raw connectivity matrices; there was also additional predictive value in computing a very dense connectivity matrix to represent the structural connectivity between all adjacent voxels in the image. This approach, known as “flow-based connectivity analysis” complemented the more standard analysis of large-scale tracts interconnecting cortical and subcortical regions of interest. Even so, brain networks and their features depend to some extent on the choice of field strength (Zhan et al., 2013c; Dennis et al., 2014), scanners (Zhan et al., 2014a), feature space (Zhan et al., 2014b), imaging acquisition parameters (Zhan et al., 2012), fiber tracking parameters (Dennis et al., 2015a), fiber tracking algorithms used to infer the trajectories of pathways in the brain (Zhan et al., 2013b, 2015a,b). Dozens of tractography algorithms are now available (Conturo et al., 1999; Mori et al., 1999; Basser et al., 2000; Lazar et al., 2003; Parker et al., 2003; Behrens et al., 2007; Aganj et al., 2011) yielding visually very different brain networks.

For this study, we adopted the tensor-based *fiber assignment by continuous tracking* (FACT) algorithm (Mori et al., 1999) to compute structural brain networks in a cohort of elderly patients with various levels of cognitive impairment (none, mild, severe). Tensor-based FACT can yield false positive fibers that may add noise to the computed network properties, but it is still one of the most widely used tractography algorithms due to it being simple and flexible. Here we propose a novel framework for network classification, with the goal of improving diagnostic classification by combining diffusion and structural MRI. We also set out to show how this new framework could be applied to networks that might contain false positive fibers (such as those derived from FACT) and used for differentiating different stages of cognition in the stages of Alzheimer's disease.

## Methods

**Figure 1:**
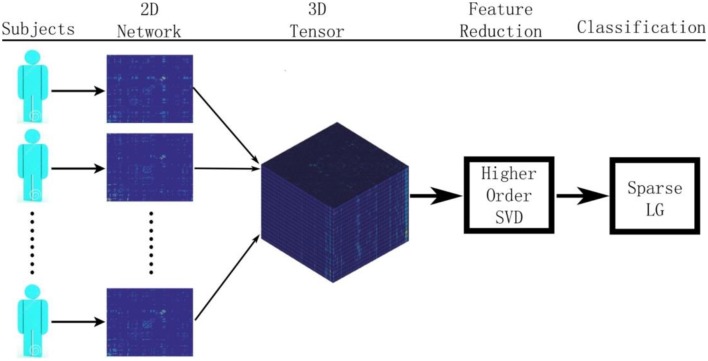
**Here we show the workflow used in this paper to classify patients based on their brain structural networks**. We model brain networks as connectivity matrices, and then stack them up, across subjects, as a 3D tensor. We then perform feature reduction and use sparse methods for diagnostic classification.

Figure 1 summarizes our proposed framework for brain network classification using higher order singular value decomposition (HO-SVD) and sparse logistic regression (Sparse LG). Its two component techniques are explained below.

### HO-SVD

Singular value decomposition (SVD) is a powerful tool for dimension reduction that is widely used in machine learning and data mining. The SVD of a matrix *X* ∈ ℝ^*n* × *m*^ is given by *X* = *U* Σ *V*^*T*^, where *U* ∈ ℝ^*n* × *n*^ and *V* ∈ ℝ^*m* × *m*^ are orthogonal matrices and Σ ∈ ℝ^*n* × *m*^ is a rectangular diagonal matrix. The diagonal entries of Σ, known as singular values, are non-negative and assumed to be in descending order.

The higher order SVD (HO-SVD) is one common generalization of SVD from matrices to tensors (De Lathauwer et al., 2000). In HO-SVD, a tensor 𝒳 ∈ ℝ^*I*_1_ × *I*_2_ × … × *I*_*N*_^ is decomposed as

$$𝒳=𝒮\times_{1}U^{\left(1\right)}\times_{2}U^{\left(2\right)}\ldots \times_{N}U^{\left(N\right)}$$

in which

1. *U*^(*k*)^ ∈ ℝ^*I*_*k*_ × *I*_*k*_^, *k* = 1, …, *N* are orthogonal matrices where the *i*th column of *U*^(*k*)^ is the *i*th *k*-mode singular vector.
2. 𝒮 ∈ ℝ^*I*_1_ × *I*_2_ × … × *I*_*N*_^ is the core tensor which is of the same size as *X*, and has the following properties:
  - For any 1 ≤ *k* ≤ *N*, let 𝒮_*i*_*k*__ and 𝒮_*j*_*k*__ be the subtensors obtained by fixing the *k*th index to *i*_*k*_ and *j*_*k*_, 1 ≤ *i*_*k*_, *j*_*k*_ ≤ *I*_*k*_, then <𝒮_*i*_*k*__, 𝒮_*j*_*k*__ > = 0 for *i*_*k*_ ≠ *j*_*k*_;
  - For 1 ≤ *k* ≤ *N*,
    $$∥{𝒮}_{i_{k}\;=\;1}∥\;\geq \;∥{𝒮}_{i_{k}\;=\;2}∥\;\geq \ldots \geq \;∥{𝒮}_{i_{k}\;=\;I_{k}}∥\;\geq \;0$$
    The Frobenius-norms ∥ 𝒮_*i*_*k*_ = *i*_ ∥, 1 ≤ *i* ≤ *I*_*k*_ are the *k*-mode singular values.

The *k*th mode singular matrix *U*^(*k*)^ can be obtained as the left singular matrix of the *k*th mode unfolding matrix of tensor 𝒳. After obtaining all *N* singular matrices *U*^(1)^ … *U*^(*N*)^, the core tensor 𝒮 is given by

$$𝒮=𝒳\times_{1}U^{\left(1\right)}{}^{T}\times_{2}U^{\left(2\right)}{}^{T}\ldots \times_{N}U^{\left(N\right)}{}^{T}$$

Inspired by the dimension reduction via SVD in the 2D case, we propose to reduce the dimensions of diffusion MRI derived brain networks, using higher order SVD (HO-SVD).

**Figure 2:**
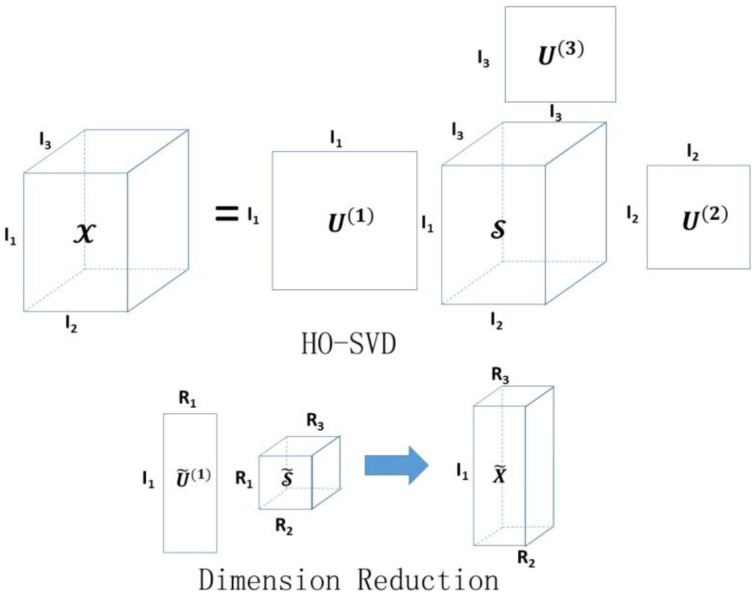
**Illustration of HO-SVD and corresponding feature reduction process**. Please refer to Section HO-SVD for the meaning of all the letters.

Similar to the matrix case, the ordering assumption for tensor singular values suggests that most of the information contained in a tensor may be expressed by the first few “components.” Let the first mode of data tensor 𝒳 correspond to the sample size *n* (i.e., *I*_1_ = *n*) and the remaining modes correspond to feature dimensions. Then, by keeping the largest *R*_1_, …, *R*_*N*_ singular values for each mode, a reduced tensor with size *n* × *R*_2_ × *R*_3_ × … × *R*_*N*_ can be obtained by

$$\tilde{𝒳}=\;\tilde{𝒮}\times_{1}{\tilde{U}}^{\left(1\right)}$$

where $\tilde{𝒮}=𝒳\times_{1}{\tilde{U}}^{{\left(1\right)}^{T}}\times_{2}{\tilde{U}}^{{\left(2\right)}^{T}}\ldots \times_{N}{\tilde{U}}^{{\left(N\right)}^{T}}$ is the core tensor with the first *R*_1_, *R*_2_, …, *R*_*N*_ singular values kept for each mode, and ${\tilde{U}}^{\left(k\right)}\in {\mathbb{R}}^{I_{k}\times R_{k}},1\leq k\leq N$. The proposed dimension reduction of the tensor is also analogous to principal components analysis (Mocks and Verleger, 1986) for a matrix input. Instead of the original tensor, we propose to use the reduced tensor $\tilde{𝒳}$ as the new input data for classification. Figure 2 illustrates the basic idea of HO-SVD and feature reduction.

### Sparse logistic regression

Let *x* ∈ ℝ^*m*^ be a sample vector and *y* ∈ {−1, +1} be a binary outcome. The logistic regression model is given by:

$$\text{Prob}(y|x)=\frac{1}{1+\text{exp}\left(-y\left(x^{T}w+c\right)\right)}$$

where *w* ∈ ℝ^*m*^ and *c* ∈ ℝ are coefficients, and Prob(y|x) is the posterior probability. Given n samples {*x*_1_, *x*_2_, …, *x*_*n*_}, the empirical logistic loss is measured by the negative log-likelihood and the average logistic loss is given by

$$\begin{array}{l} ℒ\left(w,c\right)=-\frac{1}{n}\text{log}\prod_{i\;=\;1}^{n}\text{Prob}\left(y_{i}|x_{i}\right)\\ \;=\frac{1}{n}\sum_{i\;=\;1}^{n}\text{log}\left(1+\exp\;(-y_{i}(x_{i}^{T}w+c))\right) \end{array}$$

The unknown coefficients *w* and *c* can be computed by minimizing the logistic loss, which involves a smooth convex optimization problem. However, when dimension *m* is far larger than the sample size *n*, solving the logistic regression problem is ill-posed, and the learned model may suffer from the over-fitting problem.

Sparse logistic regression embeds the feature selection into classification using the Lasso penalty (Tibshirani, 1996, 2011) which results in a sparse solution for *w*. The sparse logistic regression problem is formulated as:

$$\underset{w,c}{\min}\;ℒ\left(w,c\right)+\lambda ∥w{∥}_{1}$$

where the *l*_1_ norm of *w*, i.e., ∥*w* ∥_1_, is the Lasso penalty and λ > 0 is the regularization parameter that controls the sparsity of the solution.

## Experiments

### Subject demographics and image acquisition

We analyzed brain imaging data from 202 participants in ADNI2, the second stage of the North American Alzheimer's Disease Neuroimaging Initiative (ADNI) (http://adni.loni.usc.edu). Participant information including performance on the mini-mental state exam (MMSE) and the clinical dementia rating (CDR) are summarized in Table 1. Subjects are divided into three broad diagnostic categories based on the standard criteria outlined on the ADNI website (http://www.adni-info.org/scientists/ADNIGrant/ProtocolSummary.aspx).

- Normal Control (NC) subjects: MMSE scores between 24–30 (inclusive), CDR of 0, and non-depressed.
- MCI participants: MMSE scores between 24 and 30 (inclusive), a memory complaint, have objective memory loss measured by education adjusted scores on Wechsler Memory Scale Logical Memory II, a CDR of 0.5, absence of significant levels of impairment in other cognitive domains, essentially preserved activities of daily living, and an absence of dementia.
- AD subjects: MMSE scores between 20 and 26 (inclusive), CDR of 0.5 or 1.0, and meeting NINCDS/ADRDA criteria for probable AD.

**Table 1:** **Summary of ADNI data used in this study**.

	**AD**	**MCI**	**NC**	**Total**
Number	39	112	51	202
Age (y)	75.56 ± 9.11	71.68 ± 9.89	69.69 ± 15.43	71.92 ± 11.54
Sex	25M	71M	22M	118M

T1-weighted and diffusion MRI were acquired from each participant using 3-tesla GE Medical Systems scanners. 3D T1-weighted images were collected using spoiled gradient echo (SPGR) sequences with the following parameters: 256 × 256 acquisition matrix; voxel size = 1.2 × 1.0 × 1.0 mm^3^; TI = 400 ms; TR = 6.98 ms; TE = 2.85 ms; flip angle = 11°. 5 T2-weighted volumes with no diffusion sensitization (*b*_0_ images) and 41 diffusion-weighted volumes (*b* = 1000 s/mm^2^), were collected with the following parameters: 128 × 128 matrix; TR = 9050 ms, isotropic voxels, of size 2.7 mm; number of slices = 59; scan time = 9 min. Additional details of the protocols are available at http://adni.loni.usc.edu/wp-content/uploads/2010/05/ADNI2_GE_3T_22.0_T2.pdf. The diffusion MRI protocol for ADNI was chosen after a detailed evaluation of different protocols that could be performed in a reasonable amount of time; we reported these comparisons previously (Jahanshad et al., 2010; Zhan et al., 2013a). All T1-weighted MR and DWI images were visually checked for quality assurance to exclude scans with excessive motion and/or artifacts, and all scans were included.

### Network computation

**Figure 3:**
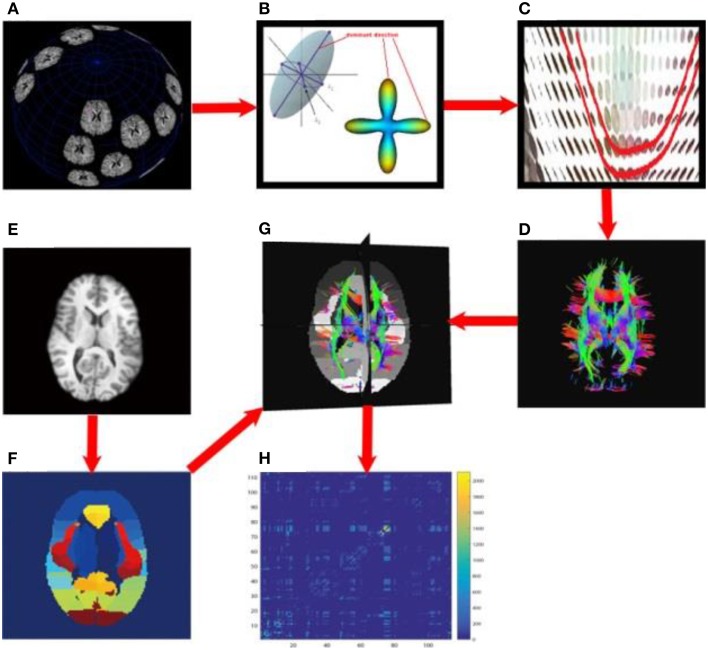
**Flowchart to compute structural brain networks**. **(A)** Diffusion MRI: the MR signal was sampled after applying gradients in a set of directions uniformly distributed on a spherical surface; **(B)** Modeling: the diffusion process was modeled using a tensor model, or by fitting orientation distribution functions, and then the dominant direction was identified; **(C)** Fiber tracking: a fiber streamline was generated, connecting as far as possible the dominant directions of neighboring voxels under some constraints (e.g., a threshold on the maximum turning angle); **(D)** whole brain tractography, tracking fibers from a set of seeds across the whole brain; here, the color indicates the fiber directions, *red* for left and right, *blue* for superior and inferior, *green* for anterior and posterior; **(E)** T1-weighted brain MRI; **(F)** brain parcellation: here we defined 113 ROIs using the Harvard Oxford Cortical and Subcortical probabilistic atlas; **(G)** aligning the whole brain tractography and 113 ROIs; **(H)** the resulting un-normalized brain network, counting the number of detected fibers connecting each pair of ROIs.

Each subject's brain network was computed with the method described in Zhan et al. (2013c). In brief, each subject's DWI was preprocessed (corrected for eddy current distortion and motion as well as removal of non-brain tissue) using the FSL toolbox (http://fsl.fmrib.ox.ac.uk/). Then, whole brain tractography was computed using tensor-based fiber assignment by continuous tracking (FACT) algorithm (Mori et al., 1999) implemented in diffusion toolkit (http://trackvis.org/dtk/). 113 cortical and subcortical regions-of-interest (ROIs) were defined using the Harvard Oxford Cortical and Subcortical probabilistic atlas (Desikan et al., 2006). For each pair of ROIs, the number of detected fibers connecting them was determined from the FACT tractography. A fiber was considered to connect two ROIs if it intersected both of them. This process was repeated for all ROI pairs, to compute a whole brain fiber connectivity matrix. This matrix is symmetric, by definition, and has a zero diagonal, i.e., we did not consider self-connections. Figure 3 illustrates the overall process to compute the brain networks.

To avoid bias in subsequent analyses, we normalized each subject's matrix by dividing each entry by its maximum value, as matrices derived from different subjects have different scales and ranges. This normalized network served as the input for the following analyses.

### Network analysis and confounds removing

For each of the 202 subjects' 113 × 113 normalized networks, we calculated standard network metrics. Five common global network measures, including *modularity* (MOD), *mean clustering coefficient* (MCC), *characteristic path length* (CPL), *global efficiency* (GLOB), and *small-worldness* (SW), were computed using the Brain Connectivity Toolbox (BCT) (Rubinov and Sporns, 2010). We used the weighted version of these measures. Definitions and mathematical equations for all of these metrics may be found at the BCT website (https://sites.google.com/site/bctnet/).

We used the generalized linear model (GLM) to remove confounds related to age and sex, across all subjects. Element-wise residual 113 × 113 networks were used as well as the residuals from the global network measures. From now on, we will refer to these networks as the “GLM-adjusted” networks and the residuals from the global network measures as GLM-adjusted network measures.

### Feature extraction

Using the 113 × 113 GLM-adjusted networks computed in Section Network Analysis and Confounds Removing, we compared three feature extraction methods:

1. *Raw features*: for each subject, the feature vector was constructed by stacking all the entries of the upper triangular portion of the matrix (as the brain matrix is symmetric). So each subject has 6328 (= 113 × 112/2) features. Thus, we obtained a matrix with 202 (subjects) by 6328 (features) as the input to Sparse LG.
2. *SVD*: We first built the raw feature matrix and then center this matrix by subtracting the means for each column. We use the top *k* principal components as the input for Sparse LG. The SVD method is essentially equivalent to PCA. Let us assume that the rows of a data matrix represent samples and the columns of data matrix represent different features. One can always use SVD to decompose an arbitrary matrix to reduce the dimensionality, by using its top k singular values and left singular vectors. When the feature columns of the data matrix are all centered, it can be easily verified that SVD is exactly the same as PCA. In our paper, we center the data matrices first, so we are essentially comparing HO-SVD with PCA.
3. *HO-SVD*: We reduced the dimension of data tensor to 202 × 15 × 15 by keeping the largest k singular values for each mode. Then, we constructed the feature vector for each subject by stacking the entries of the reduced data matrix. This constructed feature vector then serves as the input for Sparse LG.

Our empirical tests showed that the performances of SVD and HO-SVD are stable when *k* is set between 10 and 30. In this paper, we report the performances obtained after setting *k* = 15.

### Experiment design

Three comparisons, including (1) AD vs. MCI, (2) AD vs. NC, and (3) MCI vs. NC, were evaluated on the extracted features using three types of assessments:

1. *Assessment of element-wise brain connectivity matrices*: Each GLM-adjusted network cell value is compared across the different groups (AD, MCI, and NC).
2. *Assessment of global network measures*: Each of the five GLM adjusted network measures was compared across the AD, MCI, and NC groups.
3. *Assessment of feature extraction methods:* We will use the three feature extraction methods described in Section Feature Extraction to extract features from GLM-adjusted networks and conduct sparse logistic regression to evaluate the classification performance when classifying the different groups. The rows in the extracted data matrix correspond to samples, and the columns represent features. We first normalize each feature column of the input data matrix by subtracting the mean and dividing it by the standard deviation. As the outcome has an imbalanced distribution, we used undersampling techniques to mitigate the bias. Undersampling used in this paper is a special case of subsampling, and our method is essentially the bagging procedure where a bunch of models built on subsampled dataset are averaged. Different from a general subsampling strategy, undersampling requires the numbers of positive and negative samples to be the same. Thus, undersampling does not introduce any additional prior knowledge of the data distribution, so it is less likely to create classifiers that favor the majority class. For each evaluation procedure, we first randomly split data into two parts: first, 85% of the samples were used for training, and the remaining 15% of the samples were used for testing. We next ran a 5-fold cross-validation on the training data alone to select a model parameter (the LASSO parameter) and we then re-trained a model on the whole training data with the selected parameter to produce the final prediction model. We then made predictions on the test data using the final model and evaluate the performance. We repeated the training/test procedure 20 times. We report the mean and standard deviations of the classification performances including measures of accuracy, sensitivity, specificity, and the area under the curve (AUC). The Sparse LG model was implemented using the Sparse Learning with Efficient Projections package (Liu et al., 2009).

## Results and discussions

### Assessment of element-wise brain connectivity matrices

**Figure 4:**
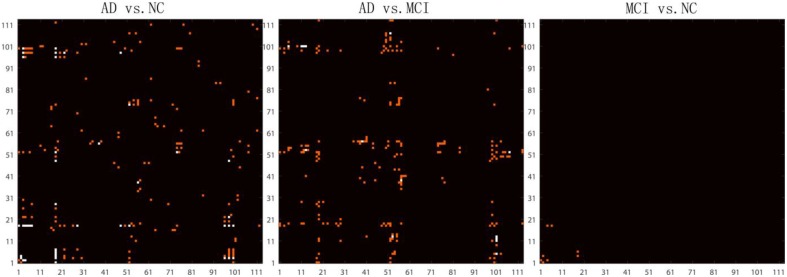
**Highlighted matrices showing Student t-test *P* maps for three diagnostic comparisons: left: AD vs. NC, middle: AD vs. MCI and right: MCI vs. NC are displayed**. Each matrix is 113 × 113, corresponding to 113 ROI connectivity pattern. The ROIs are indexed from 1 to 113. Please refer to Zhan et al. (2013c) for corresponding numbers. Each cell of the GLM-adjusted network represents the connectivity, after removing the effects of age and sex at each element. The red points in these matrices highlight the location of uncorrected *P* < 0.001. Multiple comparisons were adjusted for by Bonferroni correction and the significance threshold was set to 7.9 × 10^−6^. The white points in these matrices highlight the location of the significant differences (after Bonferroni correction) in the network cell between the groups. The greatest number of connections were different when comparing controls and AD, but no connections survive Bonferroni correction when testing differences between controls and MCI.

After removing age and sex effects, the GLM-adjusted brain network are used to estimate differences among the different diagnostic groups. To quantify these differences, we conducted Student's *t*-tests on each cell of the GLM-adjusted network for the three different tests (AD vs. NC, AD vs. MCI and MCI vs. NC). Since there are 6328 (= 113 × 112/2) cells in each GLM-adjusted network, a Bonferroni correction was adopted to account for multiple comparisons and the threshold for statistical significance was set to 0.05/6328 ≈ 7.9 × 10^−6^. Figure 4 shows the highlighted *P* map from a Student's *t*-test. Red elements in the matrices represent the connections with uncorrected *P* < 0.001. White elements in the matrices indicate connections that differ significantly between groups after Bonferroni correction. It is interesting that in the comparison between MCI and NC, there are four connections with significant uncorrected *P*-values. These connections involve brain stem, left thalamus, left putamen, left superior temporal gyrus posterior division and left hippocampus. There are considerable literatures reporting the involvement of several of these regions in degenerative neurological disorders such as Alzherimer's Disease. For example, in 2009, Simic and his colleagues reported early changes in Alzheimer's disease in the serotonergic nuclei of the brain stem, even though the brain stem would not normally appear in the set of regions with preferential atrophy in AD Simic et al. (2009) Also, the reduced volume of putamen and thalamus have been reported in Alzheimer's Disease. de Jong et al. (2008) Even so, the hippocampus is more typically one of the first brain regions to be affected by Alzheimer's Disease. Our results indicate the connection patterns among these regions may also be affected by the disease. Thus, this result deserves further investigation.

However, no significant differences were detected on an element-wise level between MCI and NC, after correction, still suggesting that it is challenging differentiate these groups based on the GLM-adjusted networks. In contrast, there were 21 significant connections for the classification task of discriminating AD vs. NC and 7 significant connections for the task AD vs. MCI. These results are consistent with our previous studies (Zhan et al., 2015b), where we found that there is an approximate order of difficulty in these differentiating tasks, with the hardest task being: MCI vs. NC > AD vs. MCI > AD vs. NC. Furthermore, comparing the *P* map between AD vs. NC and AD vs. MCI in Figure 4, we did not find any points are repeated in both *P* maps, which suggests the raw brain network cell values may not be ideal for studying of the progressive process of Alzheimer's disease. Thus, we went on to investigate network measures, in the next section.

### Assessment of global network measures

**Figure 5:**
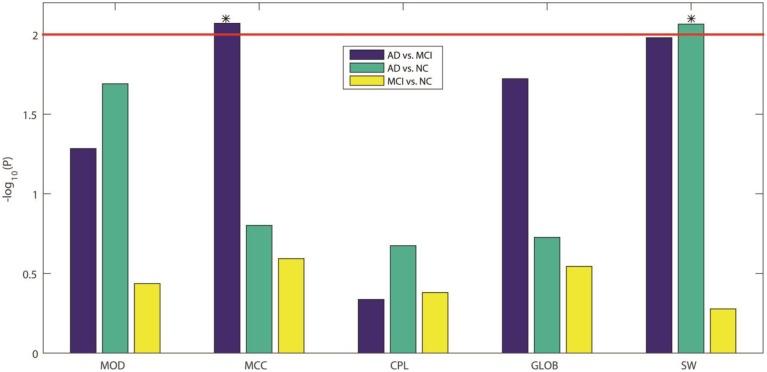
**−log_10_(P) values from our three inter-group comparisons using Student's *t*-test for each of five standard global network measures**. From left to right, the five network measures are: MOD, modularity; MCC, mean clustering coefficient; CPL, characteristic path length; GLOB, global efficiency; and SW, small worldness, respectively. The colors indicate which groups are being compared: blue for AD vs. MCI, green for AD vs. NC, and yellow for MCI vs. NC. Bonferroni correction was used to account for multiple comparisons, so the adjusted threshold is 2, indicated by the red line in the figure. Only values above the line are statistically significant given this threshold. Our results show that only MCC can differentiate AD from MCI and only SW can differentiate AD from NC.

Here we compared the five GLM-adjusted global network measures (MOD, MCC, CPL, GLOB, and SW) between the diagnostic groups. Figure 5 shows the −log_10_(P) values computed from the *t*-test between groups for these network measures, in each of the three diagnostic tasks. We again adopted a Bonferroni correction to account for the 5 comparisons in each task, so the adjusted significance threshold at the alpha = 0.05 level is 0.01 (=0.05/5). We marked this adjusted threshold with a red horizontal line [2 = −log_10_(0.01)] Our results showed that SW can be used to differentiate AD from NC while MCC can differentiate AD from MCI. As in Section Assessment of Element-wise Brain Connectivity Matrices, no measure was able to statistically distinguish between MCI and NC, which again indicates that more sensitive brain imaging features are needed to distinguish MCI from NC, at least in samples of this size.

### Assessment of feature extraction methods

Here we conducted more advanced feature extraction methods and classification techniques as described in Sections Feature Extraction and Experiment Design to better distinguish diagnostic classes. We firstly applied McNemar's test (McNemar, 1947) to confirm there are significant differences between different feature extraction methods. (Please refer to Supplementary Table for the results of the McNemar's test). Then we started to rank these feature extraction methods. Table 2 summarizes the classification performance, and Table 3 lists the Student's *t*-test *P*-values. The column *SVD > Raw* in Table 3 indicates statistical differences in classification performance of the SVD and Raw feature sets; there was no detectable difference in classification performance between these feature sets for all three diagnostic tasks. Therefore, for this particular set of tasks and this dataset, performing SVD does not improve classification performance. SVD can reduce the dimension of the data, perhaps also reducing the noise, it may still discard useful information that may be vital for classification.

**Table 2:** **Classification Performance for our three feature extraction methods**.

		**Raw**	**SVD**	**HO-SVD**
AD vs. NC	Accuracy	0.7104 ± 0.0816	0.7000 ± 0.1008	0.7125 ± 0.1020
	Sensitivity	0.6750 ± 0.1910	0.6750 ± 0.1985	0.7167 ± 0.1881
	Specificity	0.7222 ± 0.0845	0.7083 ± 0.1080	0.7111 ± 0.1134
	AUC	0.7611 ± 0.1162	0.7694 ± 0.1140	0.7806 ± 0.1117
AD vs. MCI	Accuracy	0.6288 ± 0.0454	0.6259 ± 0.0456	0.6894 ± 0.0612
	Sensitivity	0.5750 ± 0.1750	0.5417 ± 0.1418	0.6083 ± 0.1816
	Specificity	0.6329 ± 0.0484	0.6323 ± 0.0476	0.6956 ± 0.0691
	AUC	0.6195 ± 0.0997	0.6359 ± 0.0807	0.6520 ± 0.1086
MCI vs. NC	Accuracy	0.5311 ± 0.0529	0.5383 ± 0.0610	0.5734 ± 0.0533
	Sensitivity	0.5304 ± 0.0649	0.5384 ± 0.0707	0.5754 ± 0.0568
	Specificity	0.5375 ± 0.1677	0.5375 ± 0.1411	0.5563 ± 0.1489
	AUC	0.5585 ± 0.0892	0.5653 ± 0.0884	0.6104 ± 0.0777

**Table 3:** **Student's *t* test *P*-values are shown for comparing the SVD and the HO-SVD feature sets to the Raw feature set for each of the diagnostic classification tasks**.

		**SVD > Raw**	**HO-SVD > Raw**	**HO-SVD > SVD**
AD vs. NC	Accuracy	0.6393	0.4718	0.3494
	Sensitivity	0.5000	0.2456	0.2499
	Specificity	0.6734	0.6363	0.4686
	AUC	0.4101	0.2963	0.3786
AD vs. MCI	Accuracy	0.5805	0.0005	0.0003
	Sensitivity	0.7440	0.2790	0.1017
	Specificity	0.5165	0.0010	0.0009
	AUC	0.2860	0.0267	0.0554
MCI vs. NC	Accuracy	0.3473	0.0082	0.0102
	Sensitivity	0.3562	0.0125	0.0382
	Specificity	0.5000	0.3553	0.3425
	AUC	0.4051	0.0286	0.0474

*A Bonferroni correction was adopted here, to account for multiple testing. As there are four measures including Accuracy, Sensitivity, Specificity, and AUC, the corrected P threshold in each column is 0.05/4 = 0.0125. P < 0.0125 are marked in red*.

A similar result is seen when using HO-SVD in the classification task AD vs. NC. For task AD vs. NC in Table 3, both SVD and HO-SVD feature sets performed similarly to the raw feature set. One possible explanation for this could be that the AD and NC groups are the most biologically different, so they are easier to differentiate than the other two, as is evident in Table 2. The classification performance is already quite good for raw features and there is little room for improvement.

On the other hand, our proposed HO-SVD had a significant advantage in accuracy for the other two differentiation tasks. As listed in Table 3, HO-SVD performed significantly better than raw features for accuracy and specificity for AD vs. MCI; and in accuracy and sensitivity for the task, MCI vs. NC.

Brain networks derived from FACT-based tractography often include a substantial number of false positive fibers generated. Our experimental results suggest that HO-SVD is quite effective in handling feature reduction for these noisy networks, especially in the more challenging task of differentiating cognitively healthy controls from MCI.

Alzheimer's disease involves structural atrophy detectable on MRI, as well as pathological amyloid depositions and metabolic alterations in the brain. In this study, we compared the brain network properties in different stages of Alzheimer's disease using different analysis methods. In our first two assessments using element-wise brain connectivity matrices and global network measures, respectively, we were unable to differentiate the diagnostic classes MCI and NC. But while within our HO-SVD framework, the classification performance was significantly improved compared to using raw features. The choice of tractography algorithms can also affect the generated brain network, but in our previous studies (Zhan et al., 2015b), we presented a very detailed paper that was not able to detect any significant difference in classification accuracy, using brain networks generated from different tractography methods. This was extremely surprising to us, as some tractography methods lead to a much sparser representation of brain connectivity than others. But it seemed like they were all somewhat sensitive to disease effects and their accuracy was hard to distinguish even in a DTI sample of a reasonable size. In the meantime, we also conducted similar studies using different network derived from different tractography algorithms, the accuracy was also boosted by HO-SVD in compared to SVD or raw. Because of these, we only presented the result from the most common tractography algorithm, FACT, and focused our analysis on the features from the networks and classification algorithms best suited for distinguishing between the various stages of neurodegeneration. Taken together, it seems like using HO-SVD makes more difference than the tract tracing method, at least among the ones we analyzed, which were all quite well validated and widely used. Of course the possibility remains that someone will develop a better algorithm in the future.

## Conclusion

In this study, we proposed a novel framework to differentiate different stages of cognitive impairment—from no impairment in healthy controls to mild cognitive impairment and ultimately Alzheimer's disease, using diffusion MRI derived structural networks in conjunction with a sparse machine learning method. Experimental results indicate that our proposed framework performed better than more traditional methods (direct comparisons of matrix elements or singular value decomposition; SVD) in our network classification tests. Future studies will extend this framework to multi-task classification to better detect earlier stages of Alzheimer's disease, as well as including data from other modalities (anatomical MRI, PIB-PET) that may further improve classification.

### Conflict of interest statement

The authors declare that the research was conducted in the absence of any commercial or financial relationships that could be construed as a potential conflict of interest.
