# Supplementary material for: Boosting brain connectome classification accuracy in Alzheimer's disease using higher-order singular value decomposition
Source: Front Neurosci. 2015 Jul 24;9:257. doi: 10.3389/fnins.2015.00257 (PMC4513242; doi:10.3389/fnins.2015.00257)
Supplement: Supplementary file 1 [file Table1.DOCX]

Supplementary Table

The following table shows the results of applying McNemar's test on three clinical tasks. Our results indicate there are indeed significant differences between different feature extraction methods.

| McNemar test statistic (χ^2^) | Raw vs SVD | Raw vs HOSVD | SVD vs HOSVD |
| --- | --- | --- | --- |
| AD vs NC | 21.16 | 21.81 | 12.23 |
| AD vs MCI | 15.19 | 57.98 | 54.35 |
| NC vs MCI | 42.49 | 33.47 | 30.69 |
| P value | Raw vs SVD | Raw vs HOSVD | SVD vs HOSVD |
| AD vs NC | <0.001 | <0.001 | <0.001 |
| AD vs MCI | <0.001 | <0.001 | <0.001 |
| NC vs MCI | <0.001 | <0.001 | <0.001 |
